# Retention of embryonic positional identity signatures in the adult sheep tail: evidence from *HOXB13* spatial RNA expression gradients

**DOI:** 10.1038/s41598-026-42438-7

**Published:** 2026-03-02

**Authors:** Simon Horvat, Rebecca Ellenrieder, Mojca Simčič, Maša Čater, Urška Draksler, Stefan Krebs, Neža Pogorevc, Maulik Upadhyay, Viktoria Balasopoulou, Melanie Feist, Caroline C. Friedel, Ivica Medugorac

**Affiliations:** 1 Biotechnical Faculty, Department of Animal Science, University of Ljubljana, Groblje 3, Domžale, 1230 Slovenia; 2 Institute for Informatics, Ludwig-Maximilians-University Munich, Amalienstrasse 17, 80333 Munich, Germany; 3 Laboratory for Functional Genome Analysis, Gene Center, Ludwig-Maximilians-University Munich, 80539 Munich, Germany; 4 Population Genomics Group, Department of Veterinary Sciences, Ludwig-Maximilians-University Munich, Lena-Christ-Straße 48, 82152 Martinsried/Planegg, Germany; 5 Clinic for Ruminants with Ambulatory and Herd Health Services, Ludwig-Maximilians-University Munich, Sonnenstrasse 16, 85764 Oberschleissheim, Germany

**Keywords:** HOXB13, Postnatal spatial gene expression, Sheep tail length, Skin and bone, RNAseq, Positional identity, Developmental biology, Evolution, Genetics, Zoology

## Abstract

Body axis patterning in vertebrates is controlled by *HOX* genes during embryogenesis, with their expression gradients defining spatial identity along the anterior–posterior axis. While the developmental roles of *HOX* genes in axial patterning are well established, it remains unclear to what extent embryonically defined anterior–posterior *HOX* expression patterns are retained as transcriptional signatures in adult tissues. Tail length in sheep provides a model for studying the phenotypic effects of *HOX* gene variants. We used Improved Jezersko–Solčava sheep, which segregate for both tail length and *HOXB13* promoter variants, to examine genotype–phenotype associations. The *HOXB13* genotype was the main determinant of adult tail length, acting primarily by modulating caudal vertebral number. RNA‑seq and qPCR analyses revealed a clear anterior–posterior *HOXB13* expression gradient in adult tail skin and bones, consistent with retained positional patterning, with stronger distal expression in short‑tailed animals. These results provide in vivo evidence that spatially patterned *HOX* gene expression is retained in adulthood, consistent with residual transcriptional signatures of embryonic anterior–posterior identity.

**Supplementary Information:**

The online version contains supplementary material available at 10.1038/s41598-026-42438-7.

## Introduction

### Tail development and the role of *HOX* genes

Body axis patterning in vertebrates is orchestrated by the *HOX* genes. These genes belong to an evolutionarily conserved family of transcription factors that confer positional identity along the anterior–posterior axis through colinear expression during embryogenesis. Disruption in *HOX* gene regulation alters morphological developmental outcomes, underscoring their important role in defining body architecture along the vertebrate axis^1,2^. In mammals, the body axis terminates in a tail, whose length is controlled by various genetic determinants^3^. Several studies have implicated *HOX13* genes, the most posterior genes in *HOX* gene clusters, in regulating tail length^4,5^. The *HOXB13* gene was identified in 1996 by a combination of genetic and physical mapping^6^ as the last in the *HOXB* cluster. This study also demonstrated temporal and spatial colinearity of *HOXB13* expression in the main body axis of the mouse embryo. Further transgenic studies demonstrated the importance of *Gdf11* expression in trunk-to-tail development, as well as the role of *Lin28* in supporting the expansion of axial progenitors. However, this effect is eventually counteracted by the activation of *Hox13* genes^5^. In support of this result, a study showed that loss of function of *Hoxb13* in mice results in overgrowth of all major structures derived from the tail bud, including the tail. In contrast, ectopic expression of *Hoxb13* in transgenic mice led to tail truncation^7^. A recent study in mice by Hung and Wong (2024) demonstrated that Protogenin (PRTG) regulates the trunk-to-tail HOX transition, thereby facilitating proper axial extension through coordinated expression of posterior *Hox* genes, including *Hoxb13*^8^. Altogether, these findings suggest that HOXB13 functions to halt axial extension during embryonic development, thereby establishing tail length through dosage-sensitive repression of axial growth.

### *HOXB13* and genetic regulation of tail length in sheep

*HOXB13*was found to be an important regulator of tail length in sheep and was comprehensively reviewed by Kalds et al.^9^. The tail phenotype has been identified as a highly divergent trait in sheep breeds in part due to adaptation to diverse geographical and environmental contexts^10^. This makes sheep a valuable model for dissecting the genetic architecture underlying tail length. A study of Iranian sheep breeds identified *HOXB13* as one of the genes under strong selection, particularly in comparison between the thin-tailed Zel and fat-tailed Baluchi breeds^11^. A genome-wide comparison of Ethiopian and Sudanese breeds revealed a breed-specific haplotype at *HOXB13* associated with tail length^12^. Using long-read sequencing, a 168-bp insertion in the promoter near the 5′UTR of *HOXB13* was found to be enriched in long-tailed breeds^13^. In Merinolandschaf, the same insertion - mapped as 167 bp in the *HOXB13* promoter - was found tightly linked to a nonsynonymous SNP in exon 1 and identified as a likely SINE element^14^. Sequencing discrepancies in variant size (167 bp vs. 168 bp) in this study were attributed to a homopolymeric T-stretch within the SINE. A comparison of 42 long-tailed and short-tailed sheep breeds across Eurasia 13 revealed that the insertion in the *HOXB13* 5′UTR is the most significant variant influencing tail length. This finding is consistent with a genome study of five short-tailed Ovis species and the domestic Merino sheep breed, which identified the insertion as a derived variant^14^. The independent discovery of this variant across species and methods highlights its potential causal role in regulating tail length. The evolutionary and phenotypic implications of this derived allele are discussed in detail in Sect. “Tail morphology and artificial selection in domesticated sheep”.

### Tail morphology and artificial selection in domesticated sheep

The emergence of long-tailed sheep appears to coincide with post-domestication selection events^9^, with the shift toward artificial selection for increased wool production. In fine-wool breeds such as Merino or Rambouillet, this association is apparent, as such breeds frequently display both long tails and high-quality fleece^14,15^. This co-occurrence has led to the hypothesis that long tails and wool traits may have either a linked genetic basis or have been co-selected under similar artificial selection pressures^9^. Within this conceptual framework, variation at the *HOXB13* locus plays a central role in tail elongation. The derived allele (*D/D* genotype) appears to have been preferentially co-selected and is therefore predominantly found in long-tailed sheep, whereas the ancestral allele (*A/A)* is retained in short-tailed sheep as well as in mouflon^14^. This contrasts with *TBXT*, another key gene controlling vertebral identity, which has been functionally validated to drive the extreme tail truncation observed in fat-rumped sheep^16–18^. Meanwhile, it has become apparent that tail fat deposition is influenced by additional loci, notably *PDGFD* and *BMP2*, both of which also have functional associations with adipogenesis and tissue growth^19,20^. Together, these four genomic regions, *TBXT*, *HOXB13*, *PDGFD*, and *BMP2*, constitute a working model that explains a large part of tail phenotype variability in sheep as combinations of ancestral and derived alleles. Particularly, long thin-tailed sheep are hypothesized to carry a derived allele at *HOXB13* but retain ancestral variants at fat-related loci, whereas long fat-tailed sheep are expected to carry derived alleles at both *HOXB13* and at least one fat deposition locus. This model provides a possible genetic framework for explaining how natural and artificial selection have jointly shaped tail morphology in modern sheep breeds.

### The improved Jezersko–Solčava sheep: a new model to study tail genetics

**Fig. 1:**
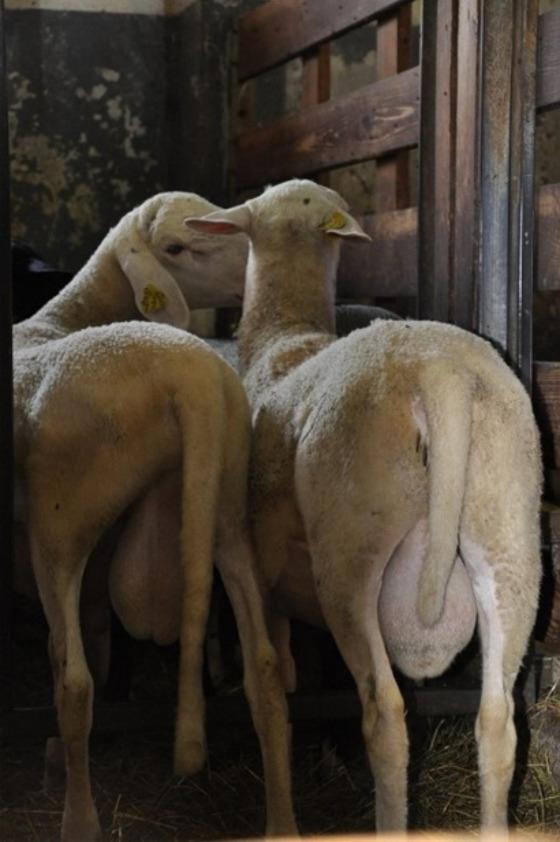
Tail length variation in the Improved Jezersko–Solčava sheep breed. Photographs depict a long-tailed ram (left) and a short-tailed ram (right), both approximately nine months old at the end of the performance test period, illustrating the natural range of tail length variation observed within the breed.

To further explore the genetic determinants of tail length in sheep, we investigated the Improved Jezersko-Solčava breed (IJS), a Slovenian local breed created by improving local Jezersko-Solčava sheep with Romanov sheep to enlarge litter size while maintaining local adaptation^21,22^. This local breed, recognized in 2021 and now with over 6,000 registered individuals^23^, presents natural variation in tail length (Fig. 1) with short, medium, and long, thin tails, making it a suitable model for dissecting the genetic basis of this trait under controlled breeding conditions^22^. Its tail length variation, together with well-documented pedigree and phenotype records, provides a valuable resource for identifying functional variants such as those in *HOXB13*, and for evaluating their effects in a population segregating for tail length.

Despite advances in identifying genetic variants affecting tail length in sheep, little is known about how key genes like *HOXB13* are regulated in adult tissues. In particular, spatial expression patterns along the anterior–posterior axis of the adult tail, as well as genotype-dependent expression effects, remain unexplored. In this study, we use the Improved Jezersko–Solčava sheep as a model breed to address this gap. By combining morphometric measurements, vertebral imaging, and spatial RNA expression analyses in both skin and bone tissues, we aim to (i) characterize the phenotypic impact of the *HOXB13* genotype on tail morphology and lumbar, sacral, and caudal vertebral numbers, (ii) define the spatial *HOXB13* expression gradient in adult tail tissues, and (iii) explore whether other tail length–associated or differentially expressed genes follow similar spatial or genotype-dependent expression patterns.

## Materials and methods

### Phenotyping and experimental design

In this study, body measurements were collected from seven cohorts of rams belonging to the Improved Jezersko–Solčava sheep breed. All animals were phenotyped at the end of the performance test period at the Test Station of the Educational and Research Center Logatec (Biotechnical Faculty, University of Ljubljana) by the same team of qualified staff. A total of 61 selected rams (Supplementary Table 1) were measured for the following morphometric traits: tail length (TL), withers height (WH), and body weight (BW). Phenotyping was carried out according to the protocol described by Eck et al^24^. at the average age of 365.89 ± 160.62 days.

Table 1 summarizes the measuring procedures for each morphometric trait. Withers height was measured with a metal measuring device designed to assess withers height, body length, and chest width in dogs used in canine sports. Tail length was measured using a custom-made wooden board containing a central groove to position the tail and a lateral scale to record its length. During measurements, the board and, consequently, the tail were held parallel to the ground to ensure consistency. Furthermore, the age of the rams at phenotyping was calculated from their birth dates, as recorded in the Central Database for Small Ruminants in Slovenia, and the phenotyping date.

**Table 1:** Measuring procedures of traits measured in live rams and in the carcass. Overview of morphometric and carcass traits measured in rams, including instruments, anatomical landmarks, and descriptive statistics.

	Trait	Measuring instrument	Measurement reference points	Mean ± SD
**Morphometric traits in live rams**	Withers height (WH), cm	Metal measuring device from dog sports	From the floor to the highest point of the withers	69.09 ± 4.96
	Tail length (TL), cm	Custom-made wooden board	From the anus to the tip of the tail	28.15 ± 5.57
	Body weight (BW), kg	Tru-Test XR3000 Electronic weigh scale		51.51 ± 13.22
**Carcass traits measured after the slaughter**	Tail length at slaughter (cm)	Analog inox beak ruler	From the first to the last caudal vertebrae	33.56 ± 7.01
	Number of sacral vertebrae	-	Counting	4.76 ± 0.70
	Length of the sacrum (cm)	Analog inox beak ruler	From the first to the last sacrum vertebrae	12.95 ± 2.18
	Number of lumbar vertebrae	-	Counting	6.90 ± 0.54
	Length of the lumbar region (cm)	Analog inox beak ruler	From the first to the last lumbar vertebrae	26.64 ± 3.67

### Slaughter procedure

The animals used in this study were not reared or slaughtered for research purposes but were rams excluded from further breeding in the national performance testing program due to failure to meet established breeding standards. Such excluded rams are always intended for slaughter and human consumption. An ear tissue sample for genotyping was collected during routine ear tagging using a GEPE Geimiplast applicator, in accordance with European animal care guidelines. All tissues used in gene expression analyses were collected postmortem. According to the Research Ethics Committee Regulation of the University of Ljubljana, Biotechnical Faculty, formal ethical approval from the National Ethics Committee for Animal Experiments was not required for this study, and the animals were sacrificed in a University Abattoir and Meat Processing Facility (Approval No. SI-37_323-02). Slaughter was performed in accordance with Regulation (EC) No 1099/2009 and national legislation, under the supervision of official veterinarians from the Slovenian Administration for Food Safety, Veterinary Sector, and Plant Protection. The animals were subjected to a mandatory pre-stunning procedure before exsanguination to avoid suffering. The experimental design and the analysis performed in the current study are in accordance with ARRIVE guidelines (https://arriveguidelines.org/). A subset of 21 rams with recorded phenotypic measurements (wither height and tail length) was selected for slaughter at an average age of 514.0 ± 148.4 days. Since measurements at slaughter were performed on a selected group of 21 animals at a later time point than the measurements taken at the conclusion of the performance test (approximately nine months of age), traits such as tail length had increased accordingly. Slaughtering was performed over seven consecutive days at the research abattoir of the Biotechnical Faculty, following a standardized protocol for all animals. Immediately after removal from the carcass, tail length was measured using an analog inox caliper, from the first to the last caudal vertebra. Following 24 h of cold storage, carcasses were bisected longitudinally along the vertebral midline using an electric saw to expose the vertebral column. The number of sacral and lumbar vertebrae was then counted, and the lengths of the sacral and lumbar regions, as well as the tail, were recorded (see Supplementary Table 2).

### Statistical analysis of morphometric traits

Analyses were conducted using R^25^. Six traits (Table 2) were analyzed with general linear models (GLM), incorporating *HOXB13* genotype (*A/A*, *A/D*, *D/D*) as a fixed effect and wither height (WH, cm) as a linear covariate to control for body size. The following linear model was applied to each trait:

**Table 2:** Least-squares means (LSM ± SE) for tail and carcass traits across *HOXB13* genotypes in Improved Jezersko–Solčava sheep. The effects of genotype and wither height on tail lengths, vertebral counts, and associated skeletal measurements.

Trait	Genotype (LSM ± SE)			*P*-value		*R* ^2^
	*A/A*	*A/D*	*D/D*	Genotype	Wither height	
Tail length at the end of performance test (cm)	24.11 ± 1.08^**a**^	27.06 ± 0.85^**a**^	31.55 ± 0.83^**b**^	< 0.0001	< 0.0001	0.50
Tail length at slaughter (cm)	28.17 ± 1.03	N/A	39.49 ± 1.08	< 0.0001	0.0037	0.78
Number of sacral vertebrae	4.81 ± 0.22	N/A	4.71 ± 0.23	n.s.	n.s.	0.03
Length of the sacrum (cm)	12.72 ± 0.61	N/A	13.19 ± 0.64	n.s.	0.0343	0.23
Number of lumbar vertebrae	6.72 ± 0.15	N/A	7.11 ± 0.16	n.s.	n.s.	0.19
Length of the lumbar region (cm)	26.20 ± 0.92	N/A	27.12 ± 0.96	n.s.	0.0042	0.38

n.s. - non-significant, LSM – least-square means, SE – standard error, R^2^ – coefficient of determination.

N/A - data not available for heterozygotes at slaughter.

1 $${Y}_{ij}=\mu+{G}_{i}+\beta\left({WH}_{ij}\right)+{\epsilon}_{ij}$$

Where *Y*_*ij*_ is the observed value of a given trait (e.g., tail length) for individual *j* with genotype *i*; *µ* is the overall mean; *G*_*i*_ is the fixed effect of *HOXB13* genotype (*A/A*, *A/D*, *D/D*); *β* is the regression coefficient for wither height *WH*_*ij*_; and *ε*_*ij*_ is the residual error.

Models were fitted separately for each quantitative trait using the *lm()* function. Type III ANOVA tables were generated using the *Anova()* function from the *car* package^26^, applying sum-to-zero contrasts (*contr.sum*) to get sums of squares. Least-squares means (LSM ± SE) for each genotype and Tukey-adjusted pairwise comparisons among genotypes were estimated using the package *emmeans*^27^. A *P*-value < 0.05 was considered statistically significant.

To further evaluate the mode of phenotypic expression of the *HOXB13* locus, genotype data were recoded into three alternative genetic models: additive (*A/A* = 0, *A/D* = 1, *D/D* = 2); dominant (*A/A* = 0, *A/D* and *D/D* = 1); and recessive (*A/A* and *A/D* = 0, *D/D* = 1). The best-fitting model was selected based on the lowest AIC (Akaike Information Criterion) and BIC (Bayesian Information Criterion) values. These are model selection criteria that balance model fit and complexity, with lower values indicating a better fit achieved with fewer parameters^28^.

To quantify the degree of dominance (*D*), the classical quantitative genetic model was applied^29^:

2 $$D=\frac{d}{a}=\frac{{\mu}_{AD}-\frac{\left({\mu}_{AA}+{\mu}_{DD}\right)}{2}}{\frac{\left({\mu}_{DD}-{\mu}_{AA}\right)}{2}}$$

where µ_AA_, µ_AD_, and µ_DD_ are the least-squares means for each genotype. Values of *D* were interpreted as approximately additive (|D| < 0.25), as partial dominance (0.25 ≤ |D| < 0.75), and as complete or strong dominance (|D| ≥ 0.75).

### Genotyping for *HOXB13* ancestral and derived allele

**Fig. 2:**
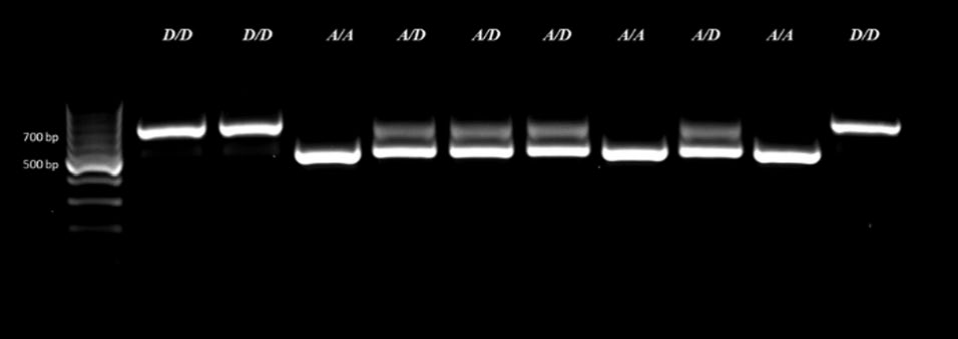
Genotyping of the 167 bp insertion in the *HOXB13* promoter region using PCR and agarose gel electrophoresis. Representative 1.5% agarose gel showing PCR amplicons distinguishing the ancestral (*A*) and derived (*D*) alleles. The *A* allele corresponds to the shorter fragment (492 bp), and the *D* allele includes the 167 bp insertion, resulting in a larger fragment (659 bp). Lanes labeled *A/A* represent homozygous ancestral genotype, *A/D* heterozygotes, and *D/D* homozygous derived genotype. A 100 bp DNA ladder (Thermo Fisher Scientific, USA) was used as a size reference.

Genomic DNA was extracted from approximately 20–30 mg of ear tissue using the E.Z.N.A.^®^ Tissue DNA Kit (Omega Bio-tek, USA) according to the manufacturer’s protocol. The purity and concentration of the extracted DNA were assessed using a NanoDrop 8000 spectrophotometer (Thermo Fisher Scientific, USA). Primers were designed to amplify across the 167 bp insertion in the *HOXB13* promoter region^13,14^ (Forward: TTTATGAGCTTCTCTCCGCCA, reverse: CACTCGGCAGGAGTAGTA). A simplified PCR-based genotyping procedure was used to distinguish carriers of the ancestral (*A*) and derived (*D*) alleles by PCR amplification and agarose electrophoresis (Fig. 2 and Supplementary Fig. 4). For the PCR reaction, we used 2 µl of DNA (10 ng/µl, diluted with water), 5 µl of FasteGene Optima Hotstart ready mix DNA polymerase (2x) (Nippon Genetics, Europe), 2 µl of H_2_O, and 0.5 µl (10 μm) of forward and reverse primer. PCR amplification was performed on a SimpliAmp thermal cycler (Applied Biosystems, USA) with the following thermal profile: initial denaturation step at 95 °C for 3 min, followed by 35 cycles consisting of 15 s at 95 °C, 30 s at 62 °C, and 30 s at 72 °C, with a final extension at 72 °C for 30 s. Amplified PCR products were separated on a 1.5% agarose gel by electrophoresis at 70 V for 45 min. Bands were visualized using an iBright CL1500 Imaging System (Invitrogen, USA). A 100 bp DNA ladder (Gene ruler, Thermo Fisher Scientific, USA) was used to estimate fragment sizes.

#### Sampling of tissues

Tissue sampling was conducted at the Training Abattoir and Cutting Plant of the Department of Animal Science, Biotechnical Faculty, University of Ljubljana. Prior to slaughter, rams were completely sheared and shaved at the designated sampling sites. All rams were humanely euthanized by electrical stunning followed by exsanguination under the supervision of an official veterinarian, as part of routine slaughter for human consumption. The animals were not slaughtered exclusively for research purposes. To minimize the risk of contamination, sterile disposable 8 mm punch biopsy tools (KAI Medical, Germany) were used for skin, muscle, and organ tissues. Each sample was immediately immersed in RNAlater^®^ (Thermo Fisher Scientific, USA) to preserve RNA integrity by inhibiting RNase activity. For bone sampling, the vertebrae were cleaned of surrounding tissues using a sterile scalpel and subsequently immersed in RNAlater^®^. Samples were then snap frozen in liquid nitrogen and transferred to − 80 °C for long-term storage. On average, no more than five minutes elapsed between exsanguination and the collection of the final tail sample.

Skin, muscle, bone, and organ tissues were collected from adult rams at predefined anatomical locations to enable downstream molecular and histological analyses. From the tail region, skin samples were collected at three defined positions along the proximal–distal axis: the tail base (at the level of the first caudal vertebra), the mid-tail (defined as half of the measured total tail length), and the tail tip (1–2 cm from the distal end of the tail). Corresponding bone samples were obtained from caudal vertebrae at matching proximal–distal positions, specifically the first caudal vertebra (tail base), a mid-caudal vertebra located approximately halfway along the caudal series, and the most distal caudal vertebra (tail tip). While bone and skin sampling locations were anatomically aligned along the proximal–distal axis, minor spatial offsets of a few millimeters between soft tissue and skeletal landmarks were unavoidable. Additional skin samples were collected from the mid-lumbar, mid-thoracic, and mid-cervical regions. Tail muscles and bones were sampled at the same three tail positions used for skin collection. Furthermore, tissue was collected from the rectum (2 cm and 14 cm from the anus), colon (5 cm and 15 cm distal to the rectum), seminal vesicles (left and right), prostate (central region), and urinary bladder (ventral and dorsal regions). This sampling strategy was designed to facilitate analysis of axial patterning, organ-specific gene expression, and structural variation across diverse tissue types and anatomical locations.

#### RNA extraction and cDNA synthesis

For skin and other soft tissues, tissue homogenization was performed using Precellys^®^ hard tissue homogenization beads (Bertin Technologies, France) in TRIzol™ reagent (Invitrogen, USA) rather than the standard lysis procedure provided in the kit. Bone tissue was crushed with a tissue pulverizer in liquid nitrogen to obtain a fine powder for subsequent RNA extraction. Total RNA was extracted using the PureLink™ RNA Mini Kit (Invitrogen, USA) following the manufacturer’s protocol, with a modification to the initial lysis step. An optional DNase treatment was also performed using PureLink™ DNase (Invitrogen, USA) to eliminate potential genomic DNA contamination. RNA purity and concentration were assessed using a NanoDrop 8000 spectrophotometer (Thermo Fisher Scientific, USA), and RNA integrity was evaluated using the LabChip^®^ GXII Touch HT system (PerkinElmer, USA). The samples had an average RNA Quality Score (RQS) of 7.1 ± 0.9.

A total of 1.5 µg of RNA from skin samples and 1.0 µg from bone samples was reverse transcribed into cDNA using the High-Capacity cDNA Reverse Transcription Kit (Applied Biosystems, USA), following the manufacturer’s protocol. In addition, no-reverse transcriptase (No-RT) controls were prepared to assess potential genomic DNA contamination. These controls followed the same procedure, except that reverse transcriptase was replaced with RNase-free water. The resulting cDNA was stored at − 20 °C until further use.

#### RNA expression analyses

For quantitative PCR (qPCR) analysis, 10 µL reactions were prepared by mixing 2.4 µL of RNase-free water, 0.3 µL of each primer (100 nM/µL; forward and reverse), 5 µL of 2× PowerUp™ SYBR™ Green Master Mix (Applied Biosystems, USA), and 2 µL of 10× diluted cDNA from skin samples and 5x diluted cDNA from bone samples. Each sample was analyzed in technical duplicates. Reactions were run on a ViiA™ 7 Real-Time PCR System (Applied Biosystems, USA) with the following thermal profile for skin samples: initial denaturation at 95 °C for 10 min, followed by 35 amplification cycles of 95 °C for 15 s, 65 °C for 1 min, and 72 °C for 30 s. A melt curve analysis was subsequently performed (95 °C for 15 s, 60 °C for 1 min, 72 °C for 30 s). For bone samples, the program was slightly modified: 50 °C for 2 min, 95 °C for 2 min, followed by 40 cycles of 95 °C for 15 s and 65 °C for 1 min. A melt curve analysis program was: 95 °C for 15 s, 60 °C for 1 min, 95 °C for 15 s. The reference genes used for normalization were *B2M* and *HMBS* for bone samples, selected based on prior gene expression studies in sheep. For the target gene *HOXB13*, two pairs of oligonucleotides (*HOXB13* F1/R1 and *HOXB13* F2/R2) were designed using the NCBI reference sequence and Primer3Plus software (Table 3). Primer specificity was validated through optimization to ensure efficient and specific binding to the cDNA template. Gene expression was analyzed by calculating ΔCt (delta Ct), −ΔΔCt, and fold change (2^−ΔΔCt^) values. To compare gene expression between genotypes, an unpaired two-tailed Student’s t-test was performed in GraphPad Prism v9.0 (GraphPad Software) on ΔCt values for each tissue. A *P*-value of < 0.05 was considered significant.

**Table 3:** Primer sequences used for RT-qPCR analysis of *HOXB13* and reference genes in sheep tissues. The table lists forward (F) and reverse (R) primer sequences used for quantitative real-time PCR (RT-qPCR). Two primer pairs (F1/R1 and F2/R2) were designed for *HOXB13* based on the NCBI reference sequence using Primer3Plus software. *B2M* and *HMBS* were used as reference genes for expression normalization.

Gene symbol	Gene name	Primer sequence	Amplicon size
*B2M*	β−2-microglobulin	F: TGTCCCACGCTGAGTTCACT	137 bp
		R: TGAGGCATCGTCAGACCTTGA	
*HMBS*	Hydroxymethyl-bilane synthase	F: CTTGCCAGAGAAGAGTGTGG	115 bp
		R: CAGCCGTGTGTTGAGGTTTC	
*HOXB13*	Homeobox B13	F1: TCGTCATGACTCCCTGTTGC	274 bp
		R1: GATCTTGCGCCTCTTGTCCT	
*HOXB13*	Homeobox B13	F2: GTCGGTGGTACAGACCTTGG	331 bp
		R2: AGATCTTGCGCCTCTTGTCC	

TaqMan Gene Expression Assays (Thermo Fisher Scientific) were used to quantify expression in skin and bone samples for the following genes: *ALOX15*, *HOXA13*, *ACSM2B*, *AGT*, *LOCL4*, and *SP8*. TaqMan qPCR reactions were performed in duplicates for each sample in 384-well optical plates using the TaqMan Universal PCR Master Mix (Thermo Fisher Scientific). Each 10 µL reaction consisted of 1.5 µL 5x diluted cDNA template, 5 µL TaqMan Universal PCR Master Mix, 0.5 µL TaqMan Gene Expression Assay, and 3 µL nuclease-free water. Plates were run on a QuantStudio 5 Real-Time PCR System (Thermo Fisher Scientific) using the manufacturer-recommended program (50 °C for 2 min, 95 °C for 20 s, followed by 40 cycles of 95 °C for 1 s and 60 °C for 20 s). The reference genes used for normalization were *B2M* and *HMBS*. For the target genes, the following TaqMan Gene Expression Assays were used: *ALOX15* (Oa04833777_m1), *HOXA13* (Oa04668307_m1), *ACSM2B* (Oa04721289_m1), *AGT* (Oa04814695_m1), *LOXL4* (Oa04673501_m1), and *SP8*, which was custom-designed.

A subset of ram samples representing distinct tail length phenotypes (short vs. long) and contrasting *HOXB13* genotypes were selected for downstream molecular validation. For qPCR and TaqMan genotyping assays, a total of 13 rams were included, seven with short tails and genotype *A/A*, and six with long tails and genotype *D/D*. From this group, six rams (three short-tailed with *A/A* genotype and three long-tailed with *D/D* genotype) were additionally selected for RNA-seq analysis. Notably, these RNA-seq samples corresponded to homozygous individuals, with *HOXB13* genotypes confirmed as *A/A* in short-tailed and *D/D* in long-tailed rams. An overview of all selected animals, including their tail length phenotype, identification number, and *HOXB13* genotype used for each analysis, is provided in Supplementary Table 3. Heterozygous animals were not included in expression analyses because these experiments were designed to contrast spatial expression extremes between homozygous genotypes; inclusion of heterozygotes, expected to show intermediate and potentially more variable expression patterns, would reduce contrast and complicate interpretation of spatial gradients.

### Roentgen examination of vertebrae

The tail examinations involved X-ray imaging of the tail bones and measuring the tail length. Tail length and the presence of vertebral malformations were documented following the methods described by Hümmelchen et al.^30^. A ruler was employed to precisely measure the tail length from the tail base to its tip. Radiographs were taken using a stationary X-ray machine (VET SYSTEM S, M.T. Medical Technology S.r.l., Italy). For X-ray imaging, 40 kV and 16 mAs were used to visualize the vertebrae. Block vertebrae were identified when the borders of adjacent vertebrae were either indistinct or completely fused. Wedge-shaped bony structures located between two vertebrae were classified as wedged vertebrae.

### RNA-seq library preparation

The isolated total RNA was further purified with Ampure XP beads (Beckman Coulter, Brea, USA) and quality-controlled on a Bioanalyzer (Agilent, Santa Clara, USA) RNA Nano chip. All samples had RIN values between 8 and 10. 100 ng of the purified total RNA was converted into RNA-seq libraries using the CorALL kit (Lexogen, Vienna, Austria) according to the manufacturer’s instructions. Briefly, 100 ng of total RNA were bound to oligo(dT) magnetic beads, eluted, and reverse transcribed with Illumina P7-tailed random primers. The 3’ends of first-strand cDNA were ligated to Illumina P5-tailed oligos. This resulted in a 5’ and 3’-tailed first-strand cDNA that was amplified with barcoded i7 and i5 index primers. Amplified libraries were purified with Ampure XP beads, analyzed on Bioanalyzer DNA 1000 chips, and quantified with Qubit dsDNA HS assay (Invitrogen, Carlsbad, USA). Equimolar amounts of barcoded libraries were pooled and sequenced on a P3 flowcell on Illumina Nextseq2000 (Illumina, San Diego, USA) in 60 bp paired-end mode.

#### RNA-seq data analysis

RNA-seq analysis pipeline details were as follows: the quality of the sequencing data was first assessed using FastQC(version 0.11.9)^31^. RNA-seq reads were aligned to the sheep reference genome (ARS-UI_Ramb_v3.0, NCBI RefSeq assembly GCF_016772045.2) and rRNA sequences using ContextMap 2 (version 2.7.9)^32^ using BWA (Burrows–Wheeler Aligner) as a short read aligner and with default parameters^33^. The number of read counts per gene and exon was quantified from the mapped RNA-seq reads in a strand-specific manner using featureCounts^34^ and gene annotations from NCBI RefSeq (GCF_016772045.2-RS_2023_10). All analysis steps were implemented and executed in a reproducible manner using the Watchdog workflow management system^35^.

Differential gene expression analysis was performed using DESeq2 (version 1.40.1)^36^ in R (version 4.3.0)^25^. To determine gene expression changes between tail tip and mid-tail vs. tail base for both *A/A* and *D/D* genotypes, we used the following design to control for differences in individual rams: ~ genotype + genotype: ram.n + genotype: location. To determine gene expression changes at each location between *A/A* and *D/D*, we used the following design to control for differences in individual rams: ~ location + location: ram.n +location: genotype. *P*-values were adjusted for multiple testing using the method by Benjamini and Hochberg for controlling the false discovery rate (FDR) implemented in DESeq2^37^, and genes with FDR ≤ 0.01 and an absolute log2 fold-change > 1 were considered significantly differentially expressed. Vulcano plots, heatmaps, and other figures were created in R. Functional enrichment analysis for Gene Ontology (GO) terms was performed using the R clusterProfiler package (version 4.8.1)^38^. For this purpose, sheep gene symbols were mapped to orthologous human genes using the R gprofiler2 package, as functional annotations are more complete for human genes. Significantly enriched (GO) terms were determined at a multiple testing adjusted *P*-value cutoff (FDR) of 0.01. Heatmaps were created using the pheatmap library in R, and clustering of genes in heatmaps was performed using Ward’s clustering criterion^39^ and Euclidean distances.

## Results

### Morphometric measurements of Improved Jezersko-Solčava sheep

**Fig. 3:**
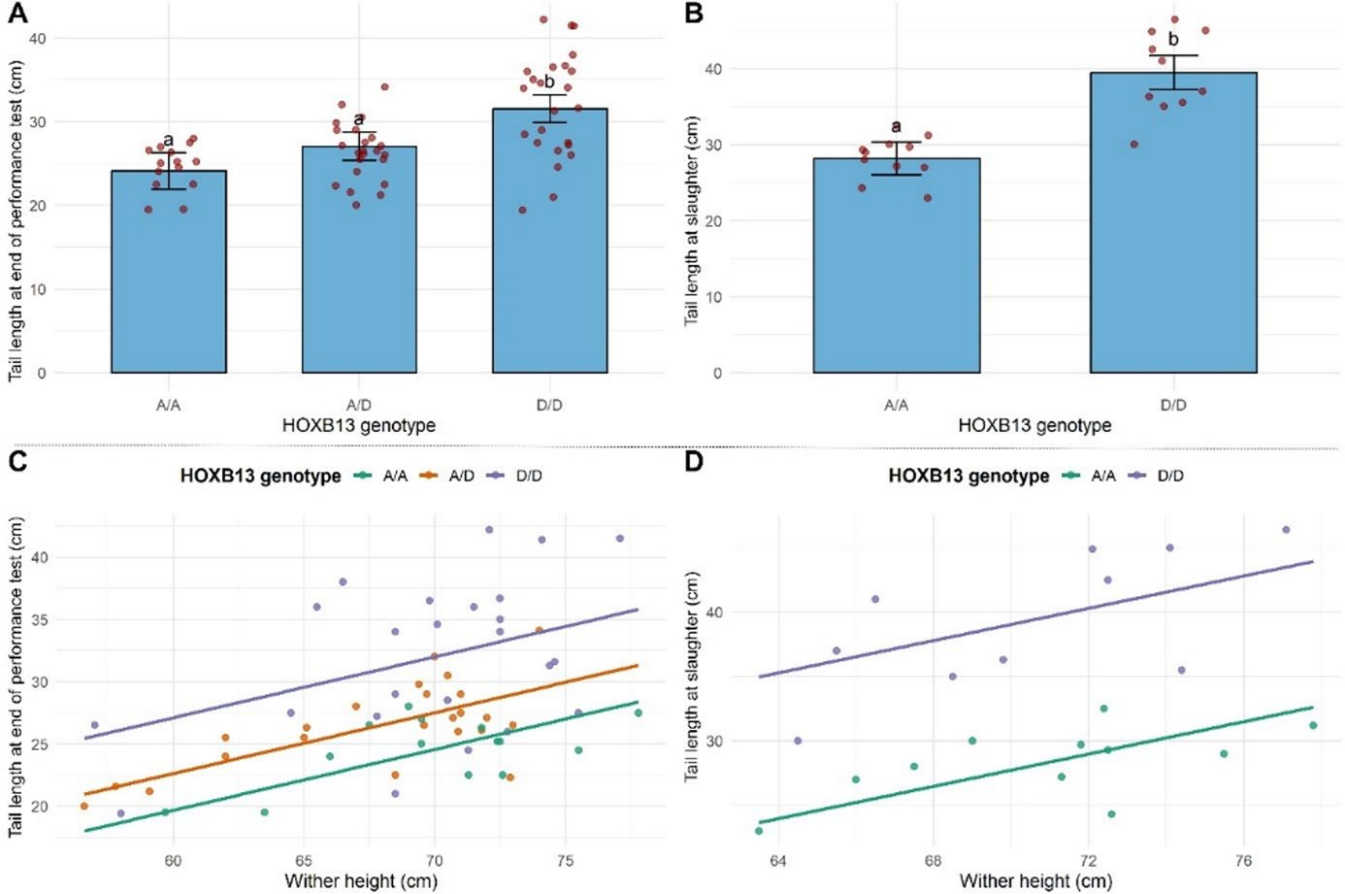
Association between *HOXB13* genotypes, wither height, and tail length in Improved Jezersko–Solčava sheep. Least-squares means ± SE for tail length measured on live animals at the end of the performance test **(A)** and after the slaughter **(B)**, adjusted for wither height. A significant positive effect of wither height on the tail length was observed across genotypes (*A/A*,* A/D*,* D/D*) in rams phenotyped (*N* = 61; *p* < 0.0001) at the end of the performance test **(C)**, and this association remained significant in a subset of rams (*N* = 21, *p* = 0.0037) assessed at slaughter **(D)**.

To assess the phenotypic effects of the *HOXB13* genotype on tail and carcass morphology, we analyzed least squares means (LSM ± SE) for six selected traits (Table 2) across genotypes (*A/A*, *A/D*, and *D/D*). A significant positive effect of wither height on tail length was observed across genotypes (Fig. 3), so our LSM analyses included wither height as a covariate (Table 2). Tail length showed a strong genotype-dependent pattern, with *D/D* animals exhibiting significantly longer tails compared to *A/A* and *A/D* animals (*p* < 0.0001). Animals with genotype *A/D* exhibited tail lengths that, on average, fell between the two homozygous genotypes, with a tendency to align closer (2.95 cm) to the mean of *A/A* animals than to that of *D/D* animals (4.49 cm). The *A/D* animals exhibited intermediate tail lengths but slightly closer to AA (2.95 cm) than to DD (4.49 cm) animals (Table 2). Thus, heterozygous animals displayed an intermediate phenotype, but with a measurable shift toward the *A/A* genotype rather than lying exactly at the midpoint between the two homozygotes. Although heterozygotes (*A/D*) were not significantly different from *A/A* animals, the ordered progression of mean tail length across genotypes (*A/A < A/D < D/D*) supports an approximately additive, gene-dosage–dependent effect of the derived allele on tail length. Wither height had a significant positive association with tail length at the end of the performance test (*P* < 0.0001), explaining half of the phenotypic variance (adjusted *R²* = 0.50, Table 2). For the selected 21 rams at slaughter, tail length was significantly affected by genotype and wither height as well (Table 2). No significant genotype effects were observed for the remaining four traits recorded in 21 rams (Table 2).

To check the phenotypic effect of the *HOXB13* variant, we compared additive, dominant, recessive, and general genotypic models for tail length, adjusting for wither height. Model comparison identified the additive model as the best fit to the data, i.e., the model with the lowest AIC or BIC value (AIC = 347.7; BIC = 356.1), followed closely by the general model (AIC = 349.2; BIC = 359.7), while the other two models showed weaker support (Supplementary Table 4). Also, the degree of dominance, *D* = −0.207, is consistent with near-additive inheritance and quantitatively reflects the slight deviation of the heterozygote mean toward the *A/A* genotype. Overall, *HOXB13* variation showed an additive mode of expression, with each copy of the derived allele incrementally increasing caudal length.

#### X-ray examination of vertebral number and tail length

**Fig. 4:**
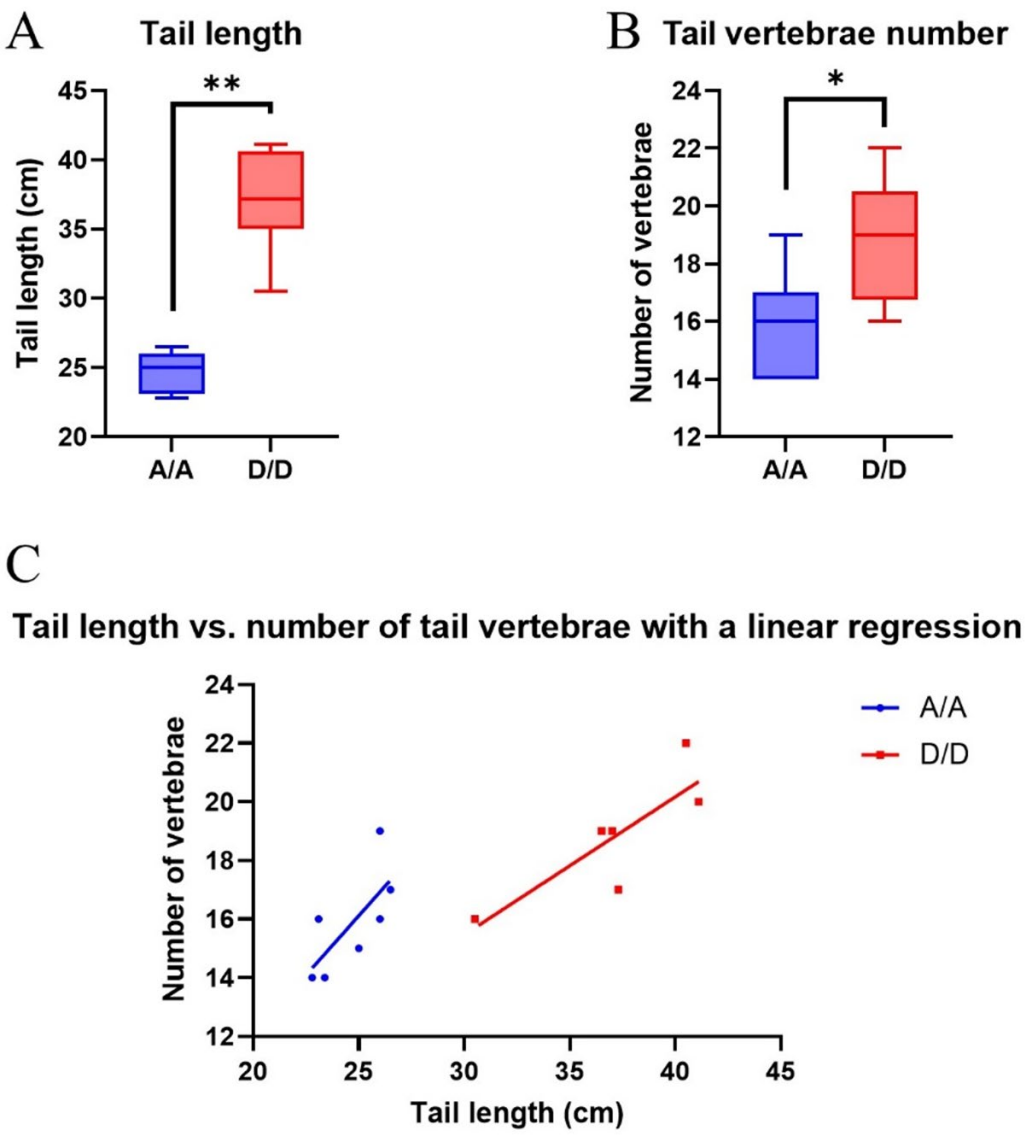
Tail length and caudal vertebrae number differences associated with *HOXB13* genotype. **(A)** Tail length comparison between ancestral (*A/A*; *N* = 7) and derived (*D/D*; *N* = 6) genotypes, showing significantly longer tails in *D/D* homozygotes (*P* = 0.0012). **(B)** Tail length is driven by an increased number of caudal vertebrae in *D/D* animals (*P =* 0.0286), with no significant difference in average vertebra length. **(C)** Linear regression analysis shows a positive correlation between total tail length and the number of caudal vertebrae across all individuals.

A subpopulation of *A/A* (*N* = 7) and *D/D* (*N* = 6) rams at slaughter (Supplementary Table 3) was also examined in detail by X-ray for the number of caudal vertebrae and their characteristics. A significant difference in tail length was observed between *HOXB13* genotypes, with homozygous carriers of the derived allele (*D/D*) exhibiting longer tails compared to homozygotes for the ancestral allele (*A/A*) (Fig. 4A). This difference in total tail length was attributed primarily to a significantly higher number of caudal vertebrae in *D/D* animals, rather than an increase in average vertebra length (Fig. 4B). Linear regression analysis further supported this relationship, demonstrating a strong positive correlation between tail length and caudal vertebral count across individuals (Fig. 4C).

**Fig. 5:**
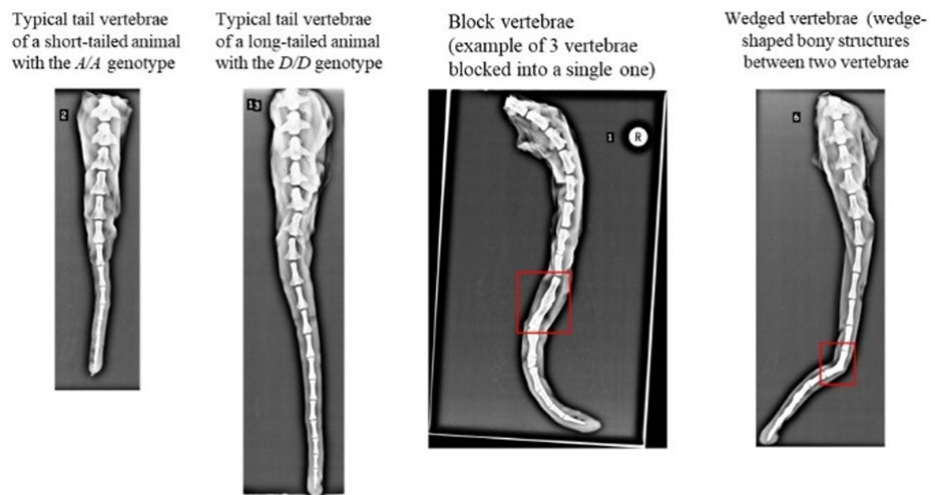
Radiographic images of sheep tails showing variation in caudal vertebral number and morphology across genotypes. Representative lateral X-ray images of tails from rams with different *HOXB13* genotypes. The images illustrate natural variation in total tail length and number of caudal vertebrae. Red boxes highlight examples of vertebral malformations (e.g., curvature or fusion anomalies). While most individuals exhibit normal vertebral alignment, a subset shows morphological abnormalities regardless of *HOXB13* genotype.

Table 4 summarizes the radiographic assessment of caudal vertebrae, vertebral malformations, and measured tail lengths across animals with different *HOXB13* genotypes. A significant difference was observed in the total number of caudal vertebrae in the tail (*P* = 0.012), but not in the number of block (*P* = 0.512) or wedged vertebrae (*P* = 0.300). Vertebral malformations, including block vertebrae (observed in 7 out of 13 animals) and a single case of a wedge vertebra, were present in both *A/A* and *D/D* genotypes, and occurred across a range of tail lengths. This suggests that these anomalies were not associated with the genotype or variation in tail length. Representative radiographic images of typical short tails (*A/A*), long tails (*D/D*), and tails exhibiting block or wedged vertebrae are shown in Fig. 5.

**Table 4:** Comparison of tail vertebrae counts and malformations between *A/A* and *D/D* genotypes. Each row represents an individual animal, identified by its ID, grouped by genotype (*A/A* or *D/D*). The table lists the total number of vertebrae in the tail and the number of malformed vertebrae categorized as either block vertebrae or wedged vertebrae.

Genotype	Animal ID	Total number of vertebrae in the tail	Including the number of vertebrae in:	
			Block vertebrae	Wedged vertebrae
*A/A*	871,932	18	7	0
	835,561	14	0	0
	835,317	14	0	0
	835,555	16	3	0
	835,507	15	3	0
	871,784	15	0	0
	871,772	15	0	0
Average		15.29	1.86	0
*D/D*	871,868	24	6	0
	871,787	16	0	0
	871,869	19	0	0
	835,402	17	3	0
	835,551	18	5	1
	835,600	19	3	0
Average		18.83	2.83	0.17
t-test (p-value)		0.012	0.512	0.3

### *HOXB13* RNA expression in the skin, colon, and rectum

**Fig. 6:**
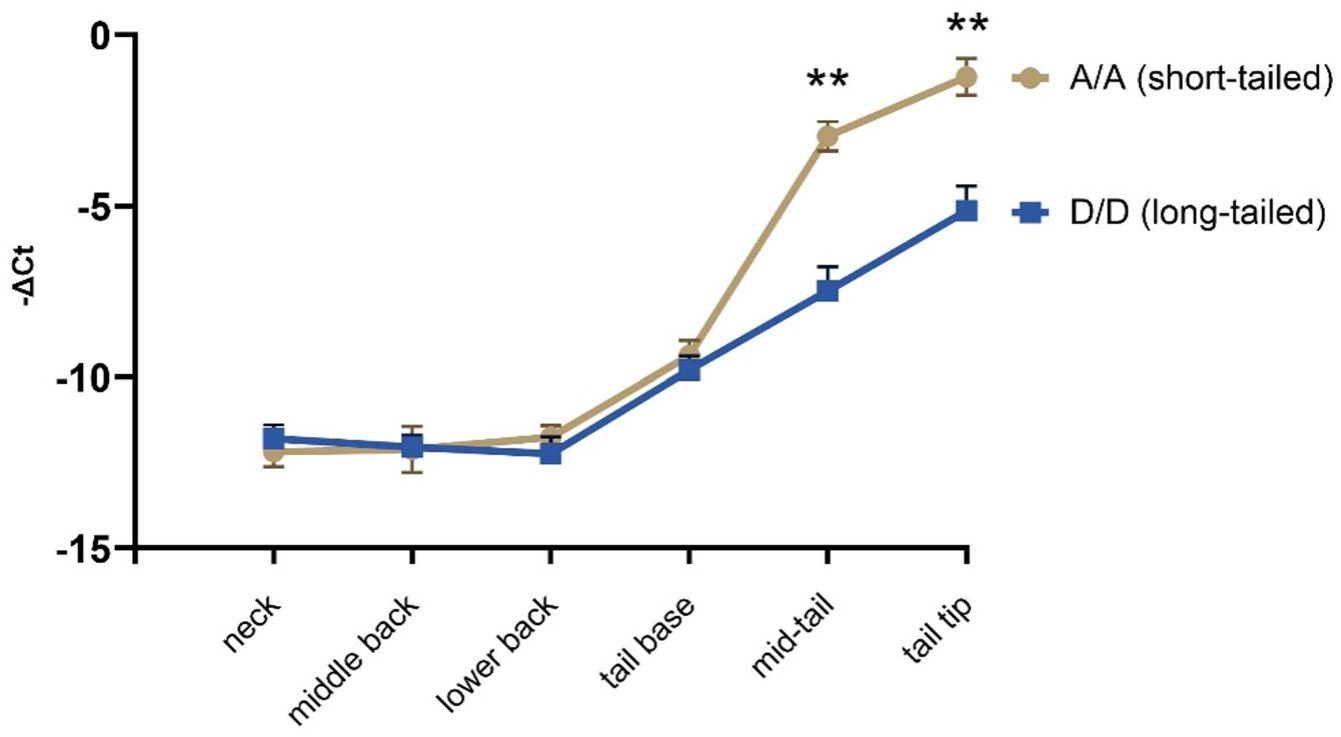
Spatial expression of *HOXB13* mRNA in skin along the anterior–posterior axis in rams of different *HOXB13* genotypes. Quantitative PCR analysis of *HOXB13* expression in skin samples collected from the mid-cervical, mid-lumbar, mid-thoracic region, tail base, mid-tail, and tail tip. Expression was normalized to reference genes (*B2M*, *HMBS*) and presented as relative expression (dCt). *HOXB13* expression was low to undetectable in anterior regions and increased progressively toward the distal tail. Expression levels were significantly higher in short-tailed rams (genotype *A/A*, *N* = 6) compared to long-tailed rams (genotype *D/D*; *N* = 6) at mid-tail and tip of the tail (*p* < 0.05). Data are shown as mean ± SD from biological replicates.

Figure 6 illustrates the spatial expression profile determined with RT-qPCR of *HOXB13* mRNA in skin samples collected along the anterior–posterior axis in rams. Expression levels were consistently low to undetectable from the mid-cervical, through the mid-thoracic, mid-lumbar, up to the tail base. A marked increase in *HOXB13* expression was observed in the mid-tail region, with the highest levels detected at the tip of the tail. Additionally, short-tailed animals (*HOXB13* genotype *A/A*) exhibited significantly higher *HOXB13* expression at the mid-tail and tail tip compared to long-tailed animals (genotype *D/D*), suggesting a genotype-dependent up-regulation of *HOXB13* in distal tail skin regions of adult sheep (*p* < 0.05).

To assess whether *HOXB13* expression patterns in the anterior-posterior direction extend beyond the skin, we examined mRNA levels in the distal colon and rectum at three positions (Supplementary Fig. 1). In contrast to the skin, where *HOXB13* expression was both spatially regulated and genotype-dependent, expression in the distal colon and rectum was uniformly high across all sampled sites, with no significant differences between *A/A* and *D/D* genotypes. Additionally, *HOXB13* mRNA expression levels in these tissues exceeded those observed in the tail tip skin.

#### RNA-seq in tail skin samples

**Fig. 7:**
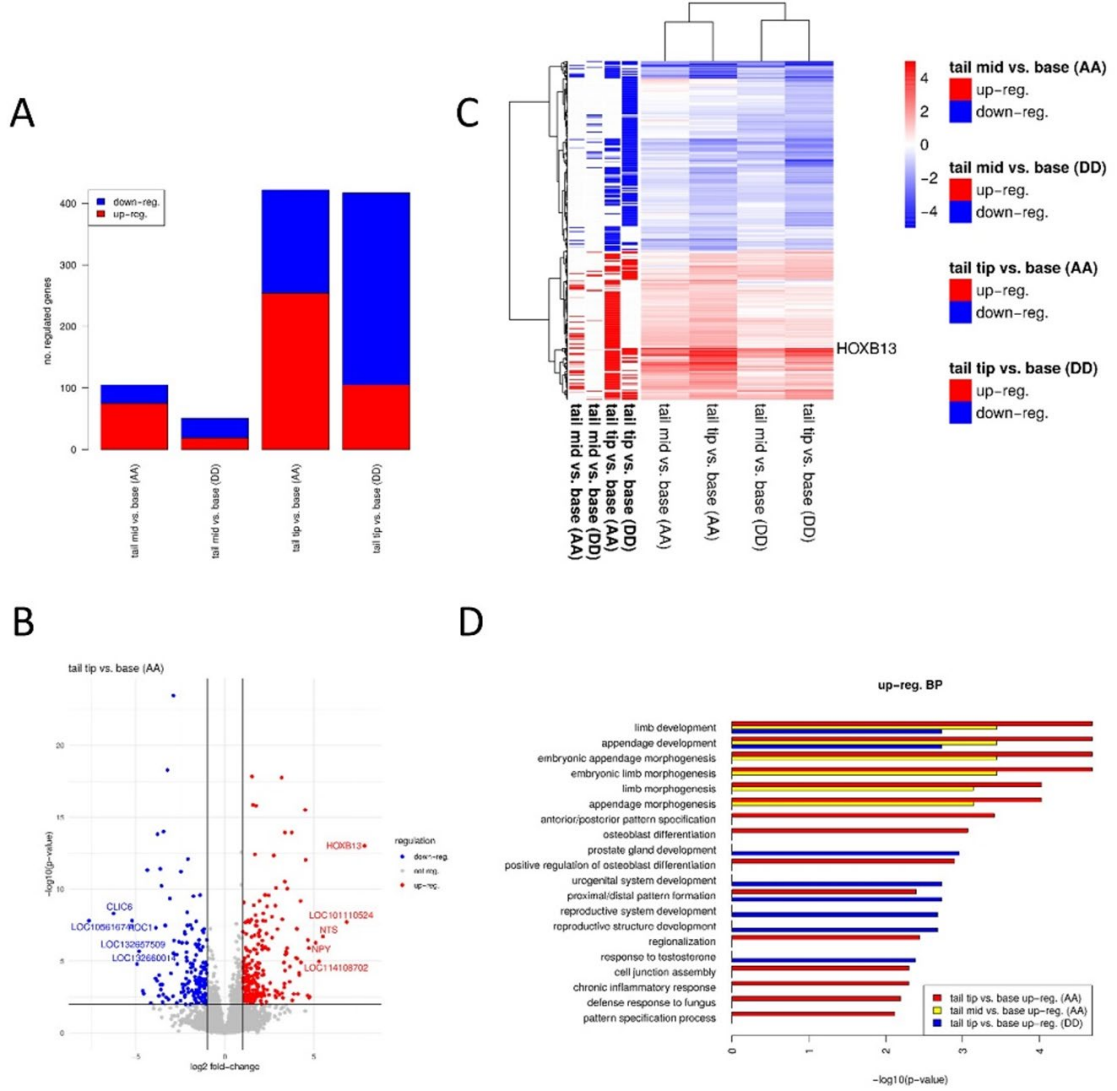
RNA-seq analysis of positional and genotypic effects in sheep tail skin. **(A)** Number of genes up- (red) and down-regulated (blue) for the comparison of mid-tail and tail tip for *A/A* and *D/D*. **(B)** Volcano plot showing log2 fold-changes and multiple testing adjusted *P*-values for the comparison of tail tip vs. base for *A/A*. Genes not significantly differentially expressed (*P*-value > 0.01 and absolute log2 fold-changes < 1) are indicated in gray. The ten most differentially regulated genes are indicated by name. **(C)** Heatmap showing log2 fold-changes in mid-tail and tip vs. base for *A/A* and *D/D* for genes differentially expressed at least once (the four right-most columns). The four columns on the left indicate whether that gene is significantly up- or down-regulated in the respective comparison. Heatmap rows and columns were clustered hierarchically using Ward’s clustering. **(D)** Functional enrichment analysis of Gene Ontology (GO) biological process (BP) terms for up-regulated genes, showing significantly enriched developmental and patterning-related processes in *A/A* and *D/D* genotypes across tail regions. Bars represent −log10(P-values).

Given the clear anterior–posterior gradient and genotype-dependent expression of *HOXB13* observed in skin by qPCR, we performed transcriptome-wide profiling using RNA-seq to explore broader gene expression patterns along the tail in both *A/A* and *D/D* genotypes. Details of the animals and samples are provided in Supplementary Table 3. The pipeline for processing RNA-seq data is described in Materials and Methods. Differential gene expression analysis on the RNA-seq data identified 105 and 51 genes differentially expressed (absolute log2 fold-change ≥ 1, FDR < 0.01) between mid-tail and base in the *A/A* and *D/D* genotypes, respectively, and 422 and 417 differentially expressed genes between tail tip and base in the *A/A* and *D/D* genotypes, respectively. Interestingly, for the *A/A* genotype, the majority of differentially expressed genes were up-regulated in mid-tail and tail tip compared to the base, while for the *D/D* genotype, the majority were down-regulated (Fig. 7A). Notably, the most up-regulated gene in the *A/A* genotype in both mid-tail and tip compared to tail base was *HOXB13*, and its expression increased from tail base to mid-tail to tip (Fig. 7B, Supplementary Fig. 2 A). In contrast, *HOXB13* was not up-regulated in the mid-tail for the *D/D* genotype (Supplementary Fig. 2B) and was less up-regulated in the tail tip (log2 fold-change 4.85, Supplementary Fig. 2 C) than even for the mid-tail in *A/A* (log2 fold-change 6.46). These RNA-seq results are fully consistent with our qPCR findings (Fig. 6), confirming both the spatial expression pattern of *HOXB13* in skin tissue and the differences between genotypes.

There was no significant difference in *HOXB13* expression between *A/A* and *D/D* at the tail base, but *HOXB13* was up-regulated at both mid-tail and tip in *A/A* compared to *D/D*. However, due to the increase in *HOXB13* expression along the tail in both *A/A* and *D/D* and the increased distance from the base to mid tail and tip for the *D/D* compared to the *A/A* genotype, these locations cannot be directly compared between genotypes. We thus focused only on changes relative to the tail base, separately for each genotype. Nevertheless, it should be noted that at the tail base, where the comparison between *A/A* and *D/D* is possible, there were only 62 genes being differentially expressed, of which 61% were up-regulated in *A/A* (Supplementary Fig. 3 A).

Figure 7C shows a heatmap with log2 fold-changes for all differentially expressed genes in mid-tail and tip compared to base for *A/A* and *D/D*, and Supplementary Fig. 3 A shows the overlap between differentially expressed genes for each comparison. This shows that genes up- or down-regulated in any condition never showed the opposite direction of regulation; however, gene expression changes between different genotypes were less similar than those between the tail tip and mid-tail within the same genotype. Furthermore, consistent with the larger number of down-regulated genes in *D/D*, down-regulated genes in *A/A* were also more frequently down-regulated in *D/D*. Vice versa, up-regulated genes in *D/D* were to a large degree also up-regulated in *A/A*, while the opposite was not the case. Moreover, the vast majority of genes (74–87%) that were differentially expressed at the mid-tail were also differentially expressed at the tail tip for the same genotype, with absolute log2 fold-changes generally increasing from mid-tail to tip.

Functional enrichment analysis for biological processes from the Gene Ontology (GO) found (embryonic) limb/appendage development as the most significantly enriched term for up-regulated genes in *A/A* in both tail tip and mid-tail compared to base (Fig. 7D). In contrast, this was only significantly enriched in *D/D* for genes up-regulated in the tail tip. Interestingly, proximal/distal pattern formation was enriched in genes up-regulated in the tail tip for both *A/A* and *D/D*, but not those up-regulated in the mid-tail. While no biological process was enriched in genes up-regulated in *D/D* in mid-tail compared to base, prostate gland and urogenital system development were enriched among genes up-regulated at the tail tip in *D/D*. The only biological process enriched among down-regulated genes at the tail tip in *D/D* was fatty acid metabolic process.

#### Validation of RNA-seq expression profiles using TaqMan qPCR in skin

**Fig. 8:**
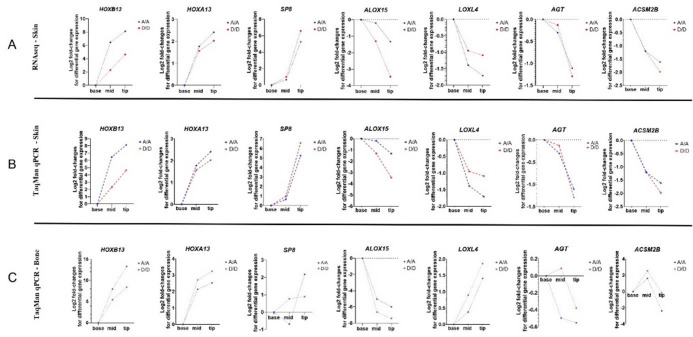
Validation of RNA-seq–derived gene expression patterns in tail skin using TaqMan qPCR. **(A)** RNA-seq log2 fold-change values for selected differentially expressed genes (*HOXB13*,* SP8*,* ALOX15*,* LOXL4*,* AGT*,* HOXA13*,* ACSM2B*) across three anatomical positions (tail base, mid-tail, tail tip) in rams with *A/A* and *D/D HOXB13* genotypes. **(B)** TaqMan qPCR validation of gene expression profiles in tail skin for the same gene set and anatomical locations. Expression changes relative to the tail base are shown as log2 fold-changes. **(C)** TaqMan qPCR analysis of the same genes in tail bone tissue at the same anatomical positions. Patterns observed in bone samples closely mirrored those found in skin, especially for *HOXB13*,* HOXA13*, and *ALOX15*. For all panels, mean expression changes are shown separately for *A/A* and *D/D* genotypes. Genes identified as up- or down-regulated in the RNA-seq data displayed concordant expression trends in qPCR validations.

To validate the RNA-seq findings, we selected a subset of genes (*HOXB13*, *SP8*,* ALOX15*,* LOXL4*,* AGT*,* HOXA13*,* ACSM2B*) for verification using TaqMan qPCR assays. These genes were selected based on their differential expression across tail regions, particularly those showing opposite expression trends between the tail base, mid-tail, and tail tip. Consistent with RNA-seq results in skin, *HOXB13* expression was verified by TaqMan qPCR in skin (Fig. 8). Other selected genes in this validation panel, i.e., *SP8*,* ALOX15*,* LOXL4*,* AGT*,* HOXA13*,* ACSM2B*, also showed the same expression pattern in the TaqMan qPCR validation assay in skin samples as was detected previously in RNA-seq analyses, confirming the validity of the RNA-seq results. The corresponding *P*-values for each spatial and genotype comparison are provided in Supplementary Table 5, highlighting which differences were significant.

#### Spatial expression of *HOXB13* and candidate genes in tail bone tissue mirrors skin RNA-seq results

To examine whether the expression of *HOXB13* and other selected differentially expressed genes from the RNA-seq analyses in skin also follows a similar spatial pattern in adult bone tissue at equivalent positions along the tail, TaqMan qPCR was performed on adult tail bone samples. The results showed the same *HOXB13* expression pattern (Fig. 8C) as observed in skin, with significant up-regulation from the tail base to mid-tail and tail-tip, and the strongest expression increase observed in the *A/A* genotype. Among the genes tested, *HOXA13* showed a spatial expression pattern similar to that of *HOXB13*, consistent with their shared role in posterior patterning. Other genes, such as *SP8*, *ALOX15*, and *LOXL4*, also showed positional changes in expression in the bone samples.

Statistical analysis of expression profiles across tissue types confirmed consistent and genotype-dependent spatial regulation of several candidate genes (Supplementary Table 5). *HOXB13* showed highly significant up-regulation from tail base to tail tip in both genotypes and in all three datasets (RNA-seq, TaqMan skin, and bone), with the strongest effect observed in the *A/A* genotype. *HOXA13* also demonstrated a consistent increase from base to tip in both skin and bone, with significantly higher expression in *A/A* compared to *D/D* animals. Other genes, including *SP8*, *ALOX15*, and *LOXL4*, showed significant spatial up-regulation in at least one dataset, particularly in *A/A* individuals. Notably, *ACSM2B* was up-regulated in the tail tip in RNA-seq and TaqMan skin data but showed limited consistency in bone. In contrast, *AGT* exhibited weaker or inconsistent spatial patterns and no genotype-dependent expression. Across all comparisons, expression differences between genotypes at the tail base were generally not significant, while significant differences were more often observed at the mid-tail and tail tip positions, especially for *HOXB13* and *HOXA13*, highlighting their likely role in tail-tip regionalization.

## Discussion

Our study suggests that variation in the *HOXB13* genotype is associated with tail length in IJS rams, primarily through differences in caudal vertebral number. Throughout this study, we use the term ‘retention’ to describe the presence of spatially patterned developmental gene expression in adult tissues, without implying continuous embryonic activity or excluding the possibility of postnatal reactivation. Rams homozygous for the derived allele (*D/D*) displayed significantly longer tails than their *A/A* counterparts, a difference supported by both morphometric measurements and radiographic imaging. Our LSM analyses indicate an approximately additive inheritance pattern, although the tail length of heterozygous animals is more skewed towards the phenotype of ancestral homozygote animals. This intermediate yet *A/A*-shifted phenotypic placement of heterozygotes suggests partial dominance of the ancestral allele and is consistent with a predominantly dosage-dependent effect of *HOXB13*. In this context, a single copy of the derived allele appears insufficient to fully recapitulate the elongated-tail phenotype observed in *D/D* animals, supporting a gradual additive phenotypic effect with allele copy number rather than a dominant or recessive mode of action.

Importantly, this elongation was not due to increased vertebral size but rather to a higher number of caudal vertebrae. These findings are consistent with recent studies in other sheep breeds that identified *HOXB13* as a major gene controlling tail length variation^9,13,14,40^. Collectively, these data support the hypothesis that variation in *HOXB13* contributes to differences in tail morphology among ovine breeds.

Body size can be roughly estimated by measuring correlated traits, such as wither height or body weight. Therefore, these measures are partly autocorrelated. The tail length showed a strong positive association with wither height, confirming that general body size contributes to variation in tail traits^24^. This association, or rather partial autocorrelation, has not been widely or systematically reported in the literature examining tail length variation, which warrants further investigation in other breeds and populations to use wither height as a covariate in statistical analyses. In our case, namely, when corrected for this covariate, *HOXB13* genotype still remained the most significant explanatory factor for tail length variation. In contrast, among other skeletal traits assessed, neither sacral nor lumbar vertebrae numbers nor lengths were significantly associated with genotype, further pinpointing the caudal region as the main site of *HOXB13*-mediated phenotypic divergence.

X-ray examinations revealed that the increased tail length in *D/D* rams was due to a higher number of caudal vertebrae rather than elongation of individual vertebrae. This finding is consistent with the known role of *HOX* genes in regulating posterior axial segmentation during embryogenesis^41,42^. In vertebrates, variation in vertebral number is determined during embryonic somitogenesis and does not arise postnatally; instead, it is classically explained by altered segmentation dynamics, including prolonged axial elongation or increased somite number, as demonstrated in multiple *Hox* mutant models^43,44^. Accordingly, the long- *versus* short-tailed phenotypes observed here are most parsimoniously interpreted as reflecting differences in developmental patterning during embryogenesis, rather than regulatory processes acting in adult tissues. Interestingly, vertebral malformations, such as block vertebrae or wedged vertebrae, were also observed but in both genotypes and did not correlate with tail length or genotype, indicating these are likely non-genetic or stochastic developmental anomalies. These findings emphasize that *HOXB13*-associated tail length variation is linked to differences in caudal vertebral number, while it does not appear to influence vertebral structural or patterning defects. This is in line with the growing body of work associating *HOX*gene variants to morphological diversity in domesticated animals^9,10,14,17^.

Our study demonstrates a clear anterior–posterior gradient of *HOXB13* mRNA expression in adult ram skin, characterized by minimal to undetectable expression from the mid-cervical through mid-thoracic, mid-lumbar, and tail base, followed by a sharp increase toward the distal tip of the tail. The pattern in the tail was also replicated in the adult ram bone tissue. To our knowledge, this is the first report demonstrating that *HOXB13* RNA expression in adult tail skin and bone follows an anterior–posterior pattern similar to that observed during embryogenesis^6,42,45^. This spatial expression pattern, peaking at the tail tip, aligns with the gene’s known role in posterior axis regulation. Moreover, the significantly higher *HOXB13* expression in short-tailed animals (*A/A* genotype) compared to long-tailed rams (*D/D* genotype) in the mid- and distal tail regions suggests that *HOXB13* activity remains transcriptionally relevant in adult tail-tip skin and bone tissues and may contribute to retention or reflection tail length phenotypes in a genotype-dependent manner. While this study was conducted in the Improved Jezersko–Solčava breed, the findings are likely relevant to sheep (genus *Ovis*) more broadly. This inference is supported by several lines of evidence: (i) both the ancestral and derived *HOXB13* variants have been associated with tail length variation across diverse domestic sheep breeds throughout Eurasia¹³; (ii) tail length is a highly heritable trait in sheep that reflects overall skeletal size, as well as the size and number of caudal vertebrae. Wild sheep are typically short-tailed with 11–14 caudal vertebrae; northern short-tailed domestic breeds show similar counts, whereas long-tailed breeds possess about 20–24 or more caudal vertebrae^46^. (iii) all examined wild short-tailed Ovis species carry the ancestral variant¹⁴; and (iv) the variants analyzed in the present study originate from two genetically and geographically distant breeds—the Russian Romanov and the Slovenian Jezersko–Solčava sheep. Nevertheless, confirmation in additional breeds and populations will be necessary to determine the full generality of these associations.

Skin was selected for transcriptome-wide profiling primarily for practical and technical reasons, including its accessibility, reproducible sampling along the proximal–distal axis of the tail, and reliable RNA yield and quality. We do not interpret skin as a proxy for paraxial mesoderm–derived skeletal tissues and explicitly acknowledge the embryological distinction between ectoderm-derived skin and mesoderm-derived vertebrae^47,48^. Accordingly, spatial gene expression patterns detected in adult skin are interpreted as descriptive transcriptional signatures along the tail axis, rather than as evidence for regulatory mechanisms governing vertebral development. Our RNA-seq analysis revealed robust spatial and genotypic variation in gene expression along the tail skin of adult rams, expanding upon the *HOXB13*-specific qPCR results. The observed anterior–posterior gene expression gradients, most pronounced in the shorter-tailed *A/A* genotype, are consistent with retained positional transcriptional patterning along the adult tail axis. Notably, *HOXB13* emerged as the most up-regulated gene in *A/A* tail tips, reflecting its established association with posterior identity during development. This result mirrors embryonic posterior *HOXB13* patterning and is consistent with the retention of embryonically defined expression signatures in adult tissues.

The asymmetry in transcriptomic regulation between genotypes, in which short-tailed *A/A* animals predominantly up-regulated positional genes while *D/D* animals showed greater down-regulation, further underscores a genotype-dependent transcriptional architecture. Moreover, the GO enrichment of limb and appendage development pathways in *A/A -* but only at the tail tip in *D/D* - suggests that positional patterning genes remain differentially active depending on the *HOXB13* allele present. However, GO enrichment analysis comparing tail-tip to tail-base expression in both genotypes revealed that biological processes associated with limb and appendage morphogenesis ranked among the most significantly up-regulated pathways.

The enrichment of biological processes related to limb and appendage morphogenesis in tail-tip vs. tail-base comparisons highlights a developmental transcriptional signature retained in adult tail tissue. Several of the up-regulated genes in the RNA-seq adult skin, *HOXA10*, *HOXA11*, *HOXA13*, *HOXC10*, and *HOXC11*, are well-characterized posterior *HOX* genes that play important roles in specifying positional identity along the anterior–posterior axis during embryogenesis^49,50^. Their retained expression in adult tail tissues suggests a lingering developmental program or regulatory memory that may contribute to the regional specialization of the tail structure.

Other regulated genes, such as *ALX4*, *DLX5*, *SP8*, *SP9*, *TBX3*, and *SHH*, are key regulators of limb bud patterning, outgrowth, and digit specification, further supporting that tail tissues share transcriptional programs with distal limb development. For instance, *SHH* is essential for posterior identity and axial elongation^51^, while *SP8/SP9* interact with Wnt and FGF pathways during distal outgrowth^52^. The up-regulation of the *SFRP2* gene, a modulator of Wnt signaling, also implicates Wnt pathway regulation in retention of positional identity or cellular differentiation in the tail tip^53^. Interestingly, *FBN2* and *ZBTB16* are involved in connective tissue architecture and skeletal patterning, which may reflect the structural remodeling or maintenance of distal caudal elements^54,55^.

Together, these findings suggest that the adult sheep tail retains transcriptional activity of key developmental regulators, particularly in distal regions, with a stronger expression signature in the ancestral short-tailed (*A/A*) genotype. This supports the hypothesis that *HOXB13*-mediated maintenance of ancestral short tail length may be accompanied or facilitated by broader (ancestral) modulation of developmental gene networks. Notably, wild sheep, such as Asian mouflon (*Ovis orientalis*), the progenitor of domestic sheep, also exhibit short tails, suggesting that the *A/A* genotype represents the conserved wild-type state. In contrast, the long-tailed *D/D* genotype likely reflects a derived condition in domestic sheep, potentially arising through disruption or attenuation of the ancestral *HOXB13*-associated regulatory program.

Although the IJS sheep is a thin-tailed breed, our transcriptomic findings of the down-regulation of fatty acid metabolism genes, particularly in long-tailed D/D animals, warrant consideration of tail fat deposition mechanisms, which are a hallmark of fat-tailed sheep. Fat-tailed sheep are physiologically well-adapted to the seasonal fluctuations and periods of feed shortage typical of arid landscapes: while they tolerate negative energy balance, they exhibit a strong anabolic response in case of a positive balance^56^. Fat-tailed sheep emerged through human-driven selection, since wild and early domestic sheep have only short, thin tails. Whereas wool lineages had already been selected by the Early Bronze Age, i.e., the 3rd millennium. BCE, selection for localized fat storage in the tail region is believed to be of a later date^57^. The selection for resilience in arid landscapes may have been triggered by the onset of a period of increased aridity known as the 4.2-thousand-year abrupt climate change event (see^58^. From a genetic perspective, several genes and/or alleles appear to participate, and candidate genes associated with fat-tailed phenotypes have been proposed in the literature (e.g.^10,20,40,59–62^), but results are equivocal. However, most wool breeds and later selected fat-tailed sheep breeds have long tails and carry *D/*D genotypes at the *HOXB13* promoter. The GO term 0006631 (fatty acid metabolic process) was significantly enriched among down-regulated genes, including *ACSM1*,* ACSM2B*,* ACSM5*,* ADIPOQ*,* ALOX15*,* ALOX5AP*,* CYP2A6*,* DAGLA*,* LEP*,* PCK1*,* PDK4*,* PLP1*,* PM20D1*,* PTGDS*,* PTGS1*, and *TNXB*. Interestingly, we did not detect differential expression of *TBXT*,* PDGFD*,* IBH*, major genetic determinants of sheep tail length previously highlighted in a comprehensive review by Kalds et al. (2022)^9^. For *TBXT*,* PDGFD*, and *IBH*, our RNA-seq analysis of adult tail skin in the IJS sheep did not reveal spatial differential expression along the anterior–posterior axis of the tail, nor did we observe genotype-dependent differences in expression between short-tailed (*A/A*) and long-tailed (*D/D*) individuals. Furthermore, several known regulators of tail development, including *BMP2*, *GDF11*, and *LIN28A*/*LIN28B*, were not differentially expressed in our dataset. Therefore, in our model, *HOXB13* was the only gene to exhibit both a pronounced spatial expression gradient and significant genotype-dependent differences in expression. This strongly suggests that allelic variation in *HOXB13* has a central role in determining tail length in the IJS sheep, although we cannot exclude contributions from other developmental regulators, particularly during earlier embryonic stages.

While our study provides some novel findings regarding the gradient expression of *HOXB13* in adult tail tissues, it has certain limitations. First, we sampled skin and bone at only three anatomical sites (tail base, mid-tail, and tail tip), providing a low-resolution overview of the anterior–posterior gradient but lacking finer resolution. Denser sampling in future studies could reveal more detailed expression patterns along the tail. Second, RNA-seq analysis was performed on skin tissue, while gene expression in bone was assessed by targeted TaqMan qPCR using a small gene set. A broader transcriptomic analysis of bone would help to fully characterize spatial regulation across tissues. Additionally, our expression data are limited to rams approximately one year of age, leaving open the question of whether the observed expression gradients persist from prenatal development or are reactivated later postnatally. Another important limitation is tissue heterogeneity. Both skin and bone contain diverse cell types, and bulk RNA methods cannot distinguish which cell populations express *HOXB13* and *HOXA13*. Future studies employing in situ hybridization, spatial transcriptomics or single-cell approaches could resolve this issue. Although our findings provide strong correlative evidence linking *HOXB13* expression gradients to genotype–phenotype differences in adult sheep tails, functional validation through in vivo transgenic models is necessary to establish causality. Furthermore, we assessed only RNA levels and did not validate protein expression or spatial localization at the tissue level, which represents a limitation of our study. It therefore remains unknown whether the observed mRNA gradients translate into corresponding protein patterns. Immunohistochemistry, in situ hybridization, or proteomic methods should be employed in future work to address this issue. Finally, our study was limited to a single European breed and a modest sample size; replication in other breeds and larger cohorts will be essential to determine whether our findings are generalizable or breed-specific.

## Conclusion

We report evidence consistent with the retention of spatial *HOX* gene expression gradients, especially *HOXB13*, in adult sheep tail tissues. Using the IJS breed, which segregates for *HOXB13* alleles and tail length, we demonstrate a clear genotype–phenotype association, mainly driven by differences in caudal vertebral number. Spatial RNA profiling of skin and bone revealed a consistent anterior–posterior *HOXB13* expression gradient, strongest at the tail tip and most pronounced in short-tailed (*A/A*) animals. This adult expression pattern mirrors embryonic *HOX* gene gradients, suggesting that elements of developmental positional-identity programs remain active or are retained in adult tissues, without distinguishing between embryonic persistence and postnatal reactivation. The IJS breed, therefore, provides a valuable model for studying the role of *HOXB13* in determining the adult tail phenotype in vertebrates. Future work should address the cellular resolution, protein-level expression, and functional relevance of these spatial expression patterns in adult tissues.

## Supplementary Information

Below is the link to the electronic supplementary material.

Supplementary Material 1

## Data Availability

RNA-Seq data were deposited into the Gene Expression Omnibus database under accession number GSE311823 and are available at the following URL: [https://www.ncbi.nlm.nih.gov/geo/query/acc.cgi?acc=GSE311823].
