# Supplementary material for: Retention of embryonic positional identity signatures in the adult sheep tail: evidence from HOXB13 spatial RNA expression gradients
Source: Sci Rep. 2026 Mar 2;16:11776. doi: 10.1038/s41598-026-42438-7 (PMC13066424; doi:10.1038/s41598-026-42438-7)
Supplement: Supplementary file 1 — Supplementary Material 1 [file 41598_2026_42438_MOESM1_ESM.pdf]

Maintenance of Embryonic Positional Identity Programs in the Adult Sheep Tail: Evidence from *HOXB13* Spatial RNA Expression Gradients

Simon HORVAT[1*], Rebecca ELLENRIEDER[2], Mojca SIMČIČ[1], Maša ČATER[1], Urška DRAKSLER[1], Stefan KREBS[3], Neža POGOREVC[4], Maulik UPADHYAY[4], Viktoria BALASOPOULOU[5], Melanie FEIST[5], Caroline C. FRIEDEL[2], Ivica MEDUGORAC[4*]

Supplementary Table 1: Cohorts, number of rams and phenotyping dates

Cohort	Number of rams	Phenotyping day
1	8	13.01.2022
2	8	01.02.2022
3	6	13.10.2022
4	9	16.12.2022
5	6	23.05.2023
6	12	15.07.2024
7	12	03.12.2024
Total	61	

Supplementary Table 2: Descriptive statistics of traits recorded in live animals and in the carcass upon slaughter

Genotype	Trait	n	Mean ± SD	Min	Max
A/A	Age at the end of the performance test (days)	14	457.71 ± 188.24	168.00	751.00
	Wither height (cm)	14	69.90 ± 4.70	59.70	77.80
	Tail length (cm)	14	24.51 ± 2.69	19.50	28.00
	Slaughter age (days)	11	525.82 ± 160.39	322.00	763.00
	Tail length at slaughter (cm)	11	28.29 ± 2.82	23.00	32.50
	Number of sacrum vertebrae	11	4.82 ± 0.75	4.00	6.00
	Sacrum length (cm)	11	12.77 ± 1.95	9.80	16.10
	Number of lumbar vertebrae	11	6.73 ± 0.47	6.00	7.00
	Lumbar length (cm)	11	26.30 ± 3.69	21.50	35.10
A/D	Age at the end of the performance test (days)	23	296.30 ± 105.79	112.00	481.00
	Wither height (cm)	23	67.81 ± 5.09	56.60	74.00
	Tail length (cm)	23	26.44 ± 3.49	20.00	34.10
	Slaughter age (days)	-	-	-	-
	Tail length at slaughter (cm)	-	-	-	-
	Number of sacral vertebrae	-	-	-	-
	Sacrum length (cm)	-	-	-	-
	Number of lumbar vertebrae	-	-	-	-
	Lumbar length (cm)	-	-	-	-
D/D	Age at the end of the performance test (days)	24	379.00 ± 162.53	138.00	687.00
	Wither height (cm)	24	69.84 ± 4.93	57.00	77.10
	Tail length (cm)	24	31.91 ± 6.27	19.40	42.20
	Slaughter age (days)	10	501.00 ± 141.50	340.00	688.00
	Tail length at slaughter (cm)	10	39.36 ± 5.39	30.00	46.40
	Number of sacral vertebrae	10	4.70 ± 0.67	4.00	6.00
	Sacrum length (cm)	10	13.14 ± 2.51	9.80	17.60
	Number of lumbar vertebrae	10	7.10 ± 0.57	6.00	8.00
	Lumbar length (cm)	10	27.01 ± 3.81	20.00	33.30

Supplementary Table 3: **Rams used for qPCR/TaqMan validation and RNAseq**. Rams in short- and long-tailed groups used for qPCR, TaqMan validation, and RNA-seq, with *HOXB13* genotypes and phenotypic measurements.

Analysis	Ram ID	*HOXB13* genotype	Tail length (cm)	Wither height (cm)
qPCR/TaqMan + RNAseq	871932	*A/A*	25.2	72.4
qPCR/TaqMan + RNAseq	871784	*A/A*	25.2	72.5
qPCR/TaqMan + RNAseq	871772	*A/A*	27.5	77.8
qPCR/TaqMan + RNAseq	835507	*A/A*	22.5	71.3
qPCR/TaqMan only	835317	*A/A*	22.5	72.6
qPCR/TaqMan only	835561	*A/A*	24.5	75.5
qPCR/TaqMan only	835555	*A/A*	26.3	71.8
qPCR/TaqMan + RNAseq	871787	*D/D*	31.5	74.4
qPCR/TaqMan + RNAseq	871869	*D/D*	36.7	72.5
qPCR/TaqMan + RNAseq	871868	*D/D*	41.4	74.1
qPCR/TaqMan only	835402	*D/D*	41.5	77.1
qPCR/TaqMan only	835600	*D/D*	36.5	69.8
qPCR/TaqMan only	835551	*D/D*	42.2	72.1

Supplementary Table 4: Comparison of alternative inheritance models for tail length in Improved Jezersko–Solčava sheep. Linear models were fitted using different genetic coding of the *HOXB13* genotype (additive, dominant, recessive, and general genotypic models), with wither height as a covariate. Lower AIC and BIC values indicate a better model fit.

**Model type**	**Genetic coding of genotype**	**Degrees of freedom**	**AIC***	**BIC****
Additive	A/A = 0, A/D = 1, D/D = 2	4	347.7	356.1
General	A/A, A/D, D/D (categorical)	5	349.2	359.7
Recessive	A/A, A/D = 0; D/D = 1	4	351.8	360.3
Dominant	A/A = 0; A/D, D/D = 1	4	360.6	369.0

*AIC (Akaike Information Criterion)

** BIC (Bayesian Information Criterion)

Supplementary Table 5: Significance of spatial and genotype-dependent gene expression differences in tail skin and bone. *P*-values for differential expression of selected genes (*HOXB13, SP8, ALOX15, LOXL4, AGT, HOXA13, ACSM2B*) across tail anatomical positions (tail base, mid-tail, and tail tip) and between genotypes (*A/A* vs. *D/D*) in three datasets: (1) RNA-seq from skin (*P*-values determined with DESeq2, see methods), (2) TaqMan qPCR from skin and (3) TaqMan qPCR from bone tissue (*P*-values determined with two-tailed t-test). Significant *P*-values ($P < 0.05$) are shown in bold.

RNA-seq skin p-values

p-value (DEseq2)	*HOXB13*	*SP8*	*ALOX15*	*LOXL4*	*AGT*	*HOXA13*	*ACSM2B*
A/A tip *vs*. base	**9.53E-14**	**0.0035**	**0.0126**	**9.76E-15**	**0.0081**	**1.57E-09**	**0.0021**
D/D tip *vs*. base	**5.91E-05**	**0.0028**	**4.08E-08**	**1.85E-06**	**0.0063**	**1.66E-11**	**1.72E-06**
A/A mid-tail *vs*. base	**3.29E-08**	0.4456	0.3757	**4.71E-06**	0.4008	**0.0010**	0.1406
D/D mid-tail *vs*. base	0.3064	0.6754	0.3558	**0.0185**	0.4286	**1.18E-07**	**0.0004**
base *D/D vs*. *A/A*	0.9924		0.5749	0.1559	0.5574	0.9678	0.4233
tip *D/D vs*. *A/A*	**3.86E-08**	0.9234	0.3009	0.9378	0.2334	0.8732	0.5870
mid-tail *D/D vs*. *A/A*	**3.69E-07**		0.6555	0.6342	0.2801	0.2605	0.6771

TaqMan qPCR skin p-values

p-value (two-tailed t-test)	*HOXB13*	*SP8*	*ALOX15*	*LOXL4*	*AGT*	*HOXA13*	*ACSM2B*
A/A tip *vs*. base	**0.0006**	**0.0006**	0.0513	**0.0006**	**0.0379**	**0.0041**	**0.0175**
D/D tip *vs*. base	**0.0022**	**0.0022**	**0.0087**	0.0931	**0.0260**	**0.0152**	**0.0152**
A/A mid-tail *vs*. base	**0.0006**	0.2075	0.6282	**0.0006**	0.2593	**0.0111**	**0.0379**
D/D mid-tail *vs*. base	**0.0130**	0.7727	0.6991	0.0649	0.9372	**0.026**	0.0649
base *D/D vs*. *A/A*	0.2162	0.4860	0.3939	0.4452	0.2343	0.4452	0.6282
tip *D/D vs*. *A/A*	**0.0023**	0.0513	0.5338	0.7308	0.2949	0.8357	0.6282
mid-tail *D/D vs*. *A/A*	**0.0012**	0.4567	0.3660	0.7308	0.0734	0.9452	0.7308

TaqMan qPCR bone p-values

p-value (two-tailed t-test)	*HOXB13*	*SP8*	*ALOX15*	*LOXL4*	*AGT*	*HOXA13*	*ACSM2B*
A/A tip *vs*. base	**0.0079**	0.1144	**0.0118**	**0.004**	0.4699	**0.0002**	0.0953
D/D tip *vs*. base	**0.0022**	0.3184	**0.0002**	**0.0276**	>0.9999	**0.0002**	>0.9999
A/A mid-tail *vs*. base	**0.0079**	0.6858	**0.0421**	0.8856	0.7576	**0.0022**	0.2525
D/D mid-tail *vs*. base	**0.013**	0.3909	**0.0022**	0.4634	0.7576	**0.0022**	0.1072
base *D/D vs*. *A/A*	0.1061	0.3023	0.4699	0.463	0.7576	0.7576	>0.9999
tip *D/D vs*. *A/A*	**0.0043**	>0.9999	0.4699	0.987	0.7576	0.7576	**0.0101**
mid-tail *D/D vs*. *A/A*	**0.0043**	**0.005**	0.2525	0.8956	0.2525	0.7576	0.2525

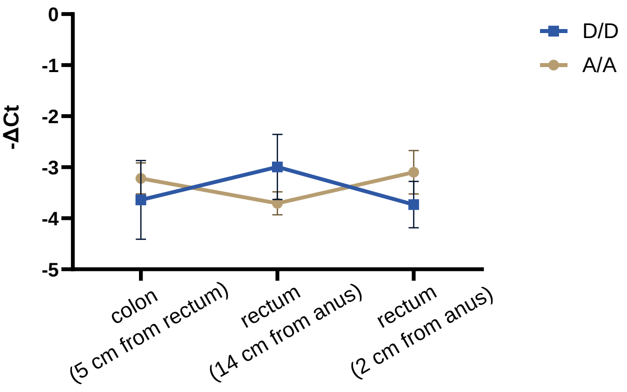

Supplementary Figure 1. HOXB13 expression in the distal colon and rectum was sampled at three positions along the anterior–posterior axis. No genotype-dependent differences or consistent spatial gradients were observed. Expression was relatively high at all sites, exceeding that in tail tip skin.

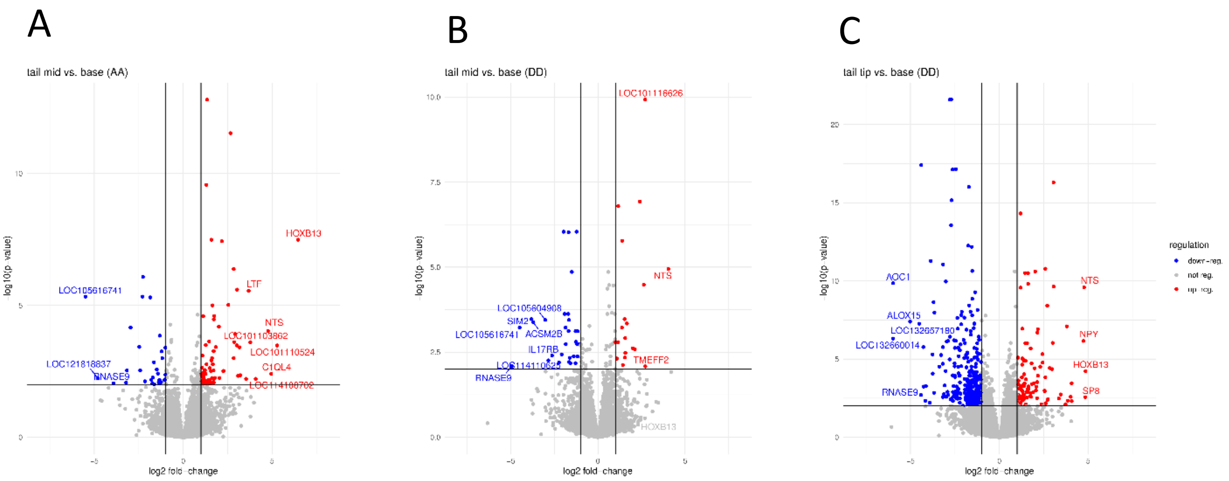

Supplementary Figure 2. RNA-seq Volcano Plots for *A/A* and *D/D* Tail Regions Along the Tail Axis. The log2 fold-changes and multiple testing adjusted *P*-values are displayed for comparisons of mid-tail vs. base for *A/A* (A) and mid-tail (B) or tip (C) vs. base for *D/D*. Genes not significantly differentially expressed (*P*-value > 0.01 and absolute log2 fold-changes < 1) are indicated in gray. The ten most regulated genes are indicated by name.

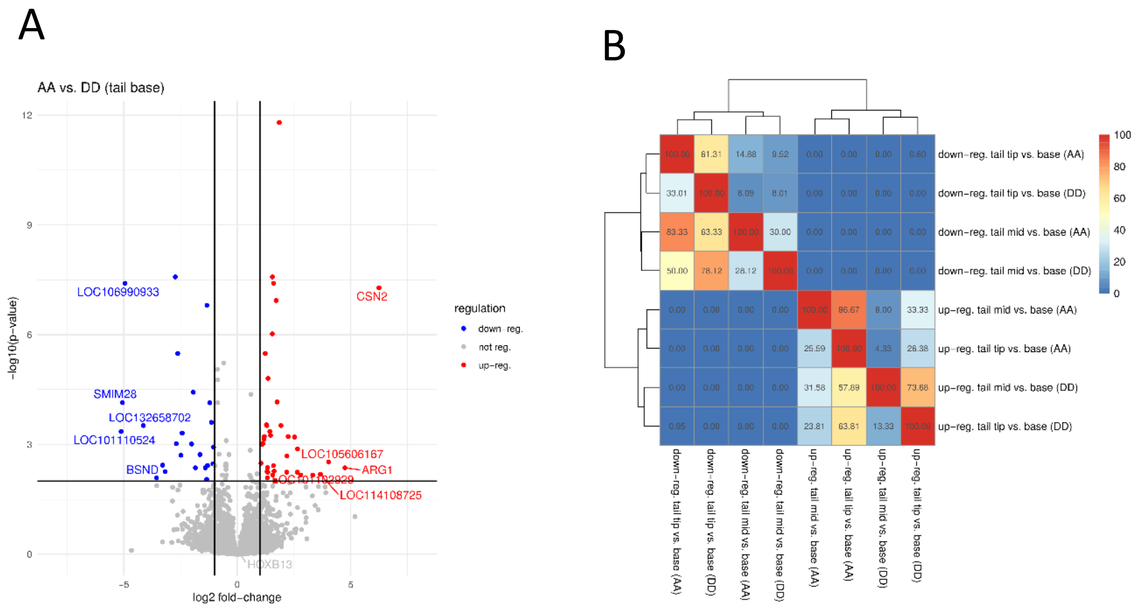

Supplementary Figure 3. Volcano Plot and Shared DEGs Across Tail regions between *A/A* and *D/D HOXB13* genotypes (A) Volcano plot showing log2 fold-changes and multiple testing adjusted p-values for the comparison of *A/A* and *D/D* at the tail base. Genes not significantly differentially expressed (p-value > 0.01 and absolute log2 fold-changes < 1) are indicated in gray. The ten most regulated genes are indicated by name. (B) Overlap between differentially expressed genes. Each cell shows the percentage of genes differentially expressed for the comparison indicated by the row, which are also differentially expressed in the comparison indicated by the column. E.g., 61.31% of genes down-regulated in tail tip vs. base for *A/A* are also down-regulated in tail tip vs. base for *D/D*. Heatmap rows and columns were clustered hierarchically using Ward's clustering criterion and Euclidean distances.

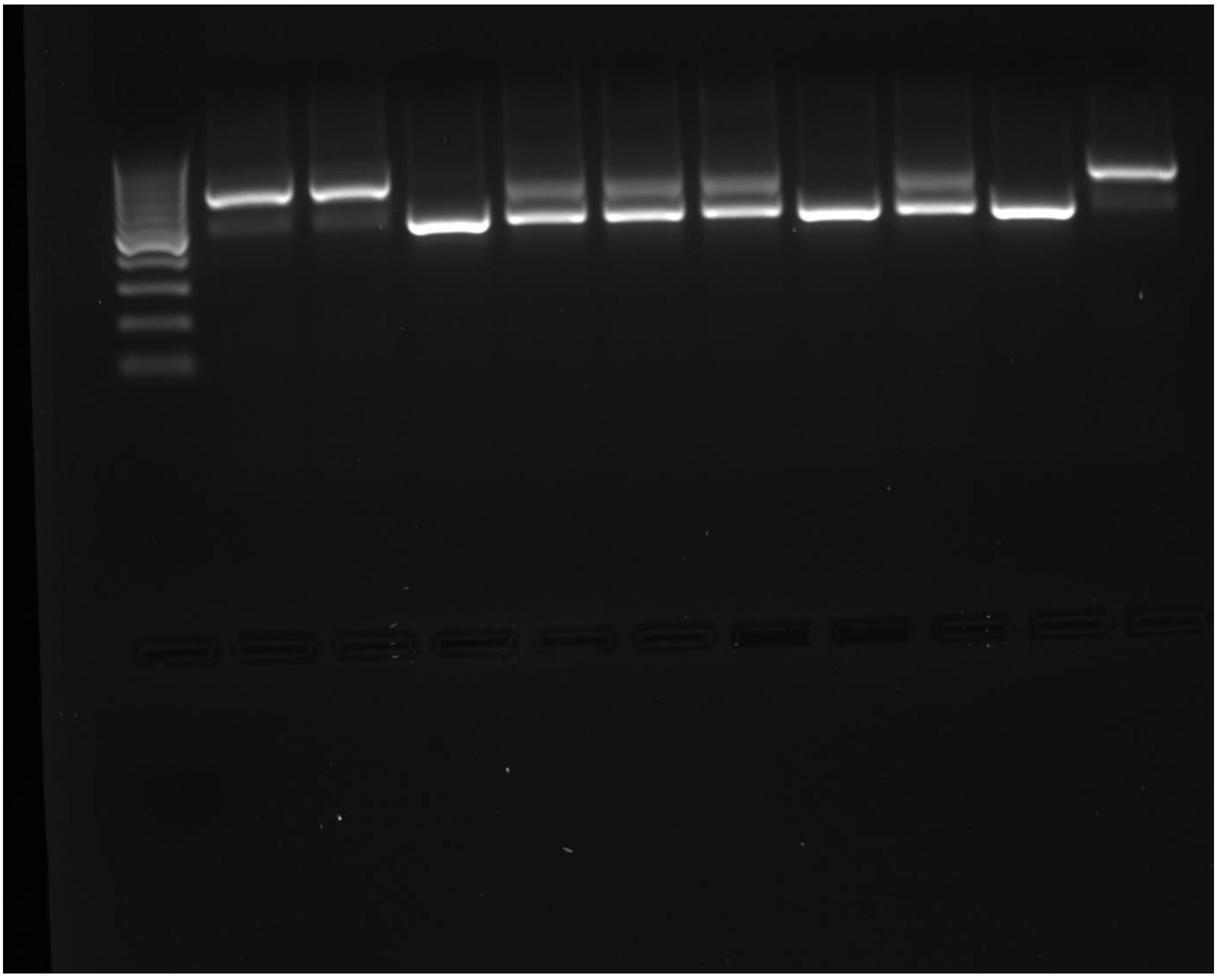

Supplementary Figure 4. Full-length, uncropped agarose gel corresponding to Figure 2. This figure shows PCR-based genotyping of the 167 bp insertion in the *HOXB13* promoter region. The gel includes all lanes and molecular weight markers as originally acquired, without cropping or contrast enhancement. Lane identities correspond to the cropped regions shown in Figure 2. A 100 bp DNA ladder (Thermo Fisher Scientific, USA) was used as a size reference. Cropped images presented in the main manuscript were generated from this gel for clarity.
